# Comparable Analysis of Acute Changes in Vascular Tone after Coffee versus Energy Drink Consumption

**DOI:** 10.3390/nu14091888

**Published:** 2022-04-30

**Authors:** Dominik Schüttler, Wolfgang Hamm, Antonia Kellnar, Stefan Brunner, Christopher Stremmel

**Affiliations:** 1 Department of Medicine I, University Hospital, LMU Munich, Marchioninistrasse 15, 81377 Munich, Germany; dominik.schuettler@med.uni-muenchen.de (D.S.); wolfgang-hamm@gmx.net (W.H.); antonia.kellnar@med.uni-muenchen.de (A.K.); stefan.brunner@med.uni-muenchen.de (S.B.); 2 DZHK (German Centre for Cardiovascular Research), Partner Site Munich Heart Alliance, 80336 Munich, Germany

**Keywords:** caffeine, coffee, energy drink, pulse wave analysis, arterial stiffness

## Abstract

Caffeinated beverages are popular throughout the world, especially due to their stimulating effects on body physiology. However, short- and long-term outcome studies have shown variable results on general health. In this pilot study, we exposed a cohort of 23 healthy individuals to 240 mg of caffeine either in the form of coffee or energy drinks and performed repetitive pulse wave analyses. This experimental approach was chosen to investigate the acute effects of caffeine consumption on vascular tone depending on the form of caffeine intake. Our data indicate that energy drinks, in contrast to coffee, might negatively impact systolic blood pressure and pulse wave velocity. This issue needs special attention in the light of cardiovascular health as the observed effects have been associated with an increased risk of cardiovascular events upon persistent exposure.

## 1. Introduction

Caffeinated beverages are among the most consumed drinks worldwide, and their stimulating effect is socially accepted [1]. Caffeine naturally occurs in beverages such as coffee and tea or is artificially added to beverages such as energy drinks. The thought of a physical performance improvement due to the positive inotropic and chronotropic effects, as well as a concentration- and memory-enhancing potential, promote the common intake of caffeine [2]. Thus, it is of high interest to investigate both the beneficial and potential adverse effects of caffeine intake. Previous analyses showed that light-to-moderate coffee consumption seemed to be associated with favorable cardiovascular outcomes [3,4]. This is further underlined by a large prospective study which found an inverse association between coffee consumption and all-cause mortality as well as cause-specific mortality [5]. In contrast to these favorable findings, concerning reports about increased cancer rates, development of arrhythmias and cardiovascular diseases after long-term consumption of caffeinated beverages were published and raise the question of whether there exists a toxic dose [6,7]. Energy drinks in particular have been linked to harmful side effects such as malignant cardiac arrhythmias, myocardial infarction and sudden cardiac death [8].

To detect physiological changes in vascular properties, a pulse wave analysis (PWA) is a commonly used non-invasive diagnostic tool, which records multiple parameters of arterial stiffness that are not assessed during routine blood pressure measurements. The European Society of Hypertension and the European Society of Cardiology included pulse wave velocity (PWV) in their guidelines to assess arterial stiffness as it is associated with increased cardiovascular morbidity and mortality and correlates with end organ damage [9].

Various studies so far have explored the effect of caffeinated beverages on cardiac function, arterial stiffness and pulse pressure, but their effects are still under debate due to conflicting findings. The discrepancies regarding blood pressure elevation and enhanced arterial stiffness range from a linear or inverse association to no observed effects, and study results vary depending on the length of exposure [10,11,12]. Moreover, there are contradictory reports in the literature as to whether the drink itself or its caffeine content are responsible for the effect on blood pressure or vascular tone [13,14]. Thus, both the precise short- as well as long-term effect of caffeinated beverages on vascular properties as well as mechanistic explanations in the sense of responsible drink components remain insufficiently explored.

In the present study, we aimed to further elucidate the acute impact of caffeine intake from either coffee or energy drinks on blood pressure behavior and the parameters of vascular tone.

## 2. Materials and Methods

We prospectively recruited 23 healthy individuals (12 female, 11 male) with an age of 31.1 (27.3, 35.2) years (median, interquartile range (IQR)) and a median body mass index of 23.7 kg/m^2^ (Table 1). Subjects with known cardiovascular disease or on daily medication were excluded from the study. Our study population consisted of regular but not excessive coffee drinkers (coffee intake was 1–2 cups/day on average) and only occasional consumers of energy drinks (<1/week). Other rich sources of caffeine (tea, guarana extracts, etc.) were inquired before enrollment and no regular consumption was found in our study population.

The cohort of this cross-over interventional pilot study was exposed to 240 mg of caffeine in two independent experiments, either by consumption of coffee or an energy drink. Therefore, each study participant took part in two independent sessions in a randomized order: In one session, volunteers consumed 750 mL of a commercial energy drink (containing 32 mg caffeine/100 mL (0.03%), 0.4% taurine, 11 g/100 mL sugar according to the manufacturer’s information). In the other session, study participants consumed three cups (40 mL/cup) of coffee (containing 80 mg caffeine/cup according to the manufacturer’s information). Coffee was brewed using three sealed espresso capsules with a constant extraction time providing altogether 120 mL. Beverages were then consumed within 15 min. There was an interval of at least 48 h between the two sessions. We urged study participants to avoid any other caffeinated beverages or alcohol for 48 h before each session.

During the sessions, pulse wave parameters were collected with a BR-102 plus pulse wave analyzer (PWA) (Schiller, Germany) in a seated resting position, and volunteers were encouraged not to talk or move. After baseline PWA recordings, the respective beverage was then consumed within approximately 15 min. In humans, 99 percent of caffeine is absorbed within 45 min of ingestion and peak blood concentrations can be detected between 15 and 120 min after oral ingestion [15]. We thus repeated pulse wave analyses 45 min after the consumption of the coffee and energy drinks. To exclude potential volume effects of drinks on pulse wave parameters, we added a third experimental cohort of 4 study participants (2 male, 2 female), who drank 750 mL of tap water. PWA was performed at baseline and 45 min after water intake.

All data were collected and analyzed by the study investigators at the Department of Cardiology at the University Hospital Munich (LMU). The study protocol was approved by the local ethics committee (“Ethikkommission der Medizinischen Fakultät der LMU München”, project no. 370-16). Informed consent was obtained from each patient, and the study protocol conformed to the ethical guidelines of the Declaration of Helsinki.

Statistics were calculated with GraphPad software Prism 9 (GraphPad, La Jolla, CA, USA). Results are presented as median and IQR. Due to limited sample sizes, we did not assume normality. P values were calculated by paired sample Wilcoxon tests and corrected for multiple comparisons using the Holm–Sidak method; *p* < 0.05 (*), *p* < 0.01 (**), *p* < 0.001 (***).

## 3. Results

**Figure 1:**
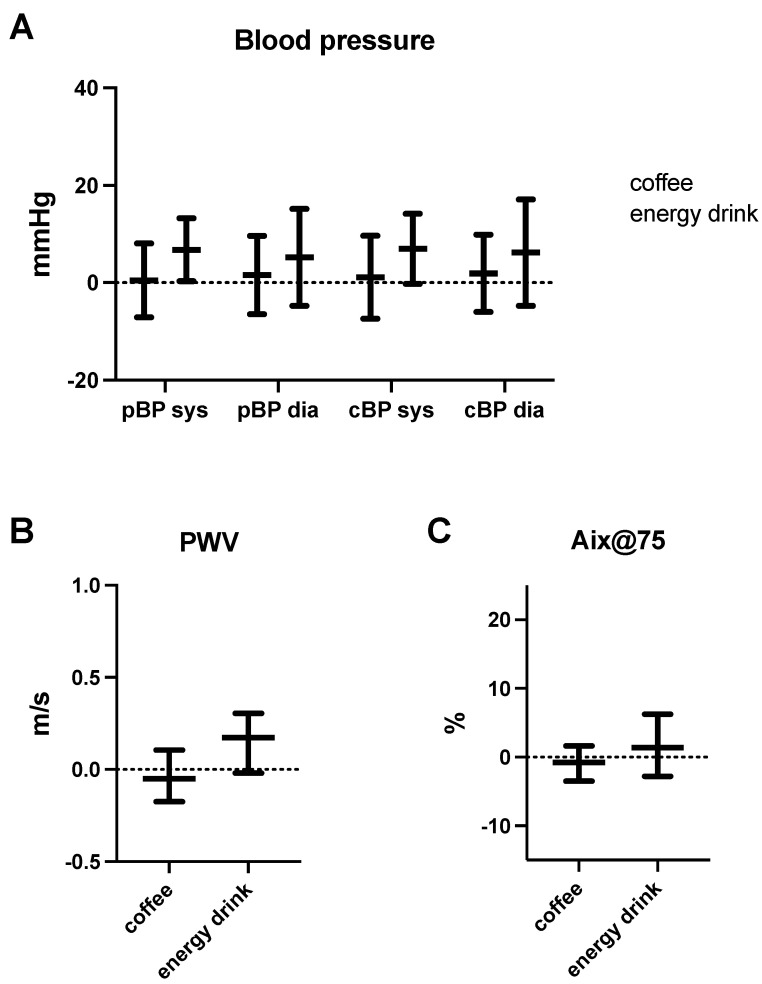
Hemodynamic parameters after caffeine consumption. Effect of energy drink and coffee consumption on systolic (sys) and diastolic (dia) peripheral (pBP) as well as central blood pressure (cBP) (**A**), pulse wave velocity (PWV) (**B**) and augmentation index (Aix) (**C**). Dots and triangles represent individual values.

Baseline heart rate was between 75–80 bpm in all experimental groups, and we did not observe any significant changes after caffeine exposure. While blood pressure remained unaffected by coffee consumption (Table 2 and Table 3; Figure 1), we detected an increase in peripheral (+6.35 mmHg) as well as central systolic blood pressure (+7.00 mmHg) after energy drink consumption (Table 2 and Table 3; Figure 1). Yet, the observed effects did not reach significance after correction for multiple testing (Table 3). Diastolic blood pressure remained unaffected.

Pulse wave velocity did not change after coffee intake but increased by 0.17 m/s after energy drink consumption (Table 2 and Table 3; Figure 1). Again, statistical significance was lost after correction for multiple testing. Other pulse wave parameters, such as the augmentation index (Aix) or augmentation pressure (AugP), were unchanged after any form of caffeine intake. Since the absolute volume of coffee (120 mL) was lower compared to energy drinks (750 mL) in our experiment, we added a third control group to exclude volume-associated effects with an intake of 750 mL water. As opposed to our energy drink group, we were not able to detect a significant change for any of the recorded parameters (Table 2).

## 4. Discussion

In this pilot study, we found strong indications that energy drink consumption leads to an increase in systolic blood pressure, as well as pulse wave velocity. However, the observed effects did not reach statistical significance after correction for multiple testing due to our small sample size. Consistent with preceding studies, diastolic blood pressure was not affected [16]. The observed effects on blood pressure and PWV were neither caused by the caffeine content of 240 mg alone as coffee had no effect on these parameters, nor was it caused by the pure volume load as our water control group demonstrated. Thus, potential effectors beyond caffeine might have been other components of energy drinks such as taurine or sugar. While sugar has been associated with an increase in blood pressure in previous studies, taurine seems to have an opposite effect [17,18,19].

Independent of the underlying biophysiological mechanism, systolic blood pressure and pulse wave velocity have clearly been associated with cardiovascular health. Our study supports previous investigations that suggest a negative impact of energy drinks on cardiovascular health due to elevated blood pressure and potential proarrhythmic effects [8]. Higher pulse wave velocities as observed in our study are direct markers of increased arterial stiffness. Although values remained below pathological thresholds in our acute setting, the long term use of energy drinks alone or in combination with other risk factors might become relevant for arterial stiffness as an increase in these parameters is associated with adverse outcome, especially upon long-term exposure [9,20]. In contrast to PWV and systolic blood pressure, Aix showed no alterations. As Aix is only a surrogate parameter for vascular tone, which is influenced by other vascular and hemodynamic properties, it is not surprising that the observed minor changes in arterial stiffness did not translate to Aix [21].

Our study might be limited by the fact that subject volunteers could not be blinded to treatment. In addition, we did not measure caffeine blood concentration so we might have missed the window of peak blood concentration during our recordings.

## 5. Conclusions

Changes in systolic blood pressure and PWV require special attention and larger studies will be needed to identify the causative component beyond caffeine itself. Our pilot study indicates a negative impact of energy drinks on vascular stiffness; however, validity is limited by the small sample size. Nevertheless, our data clearly underline the usefulness of further investigation and pave the way for larger studies. In addition, even though short-term effects were only mild, they might become more relevant in the context of habitual long-term exposure. Future long-term head-to-head comparisons between coffee and energy drinks should address the question as to whether a stimulating effect can be achieved without negative side effects, as assumed for energy drink consumption.

## Figures and Tables

**Table 1:** Baseline characteristics.

	Total (23)	Female (12)	Male (11)
Age	31.1 (27.3, 35.2)	30.6 (26.1, 34.9)	31.1 (28.7, 35.2)
Height (cm)	170 (167.5, 182,5)	167.5 (164.0, 170.0)	183 (179.0, 185.0)
Weight (kg)	70 (62.0, 85.0)	62 (60.0, 72.0)	84 (70.8, 87.5)
BMI (kg/m^2^)	23.7 (21.7, 26.0)	22.8 (21.4, 25.5)	25.7 (22.4, 26.0)

All values are presented as median (IQR). BMI, body mass index.

**Table 2:** Hemodynamic parameters before and after caffeine consumption.

**Coffee (240 mg Caffeine, 120 mL)**		
	**Coffee**	
	**Before**	**After**
**HR (bpm)**	79.6 (72.3, 84.8)	76.4 (72.0, 83.1)
**pBP sys (mmHg)**	125.8 (115.8, 132.0)	125.4 (117.0, 131.4)
**pBP dia (mmHg)**	80.9 (68.1, 91.2)	83.7 (72.7, 88.7)
**pBP mean (mmHg)**	98.3 (86.2, 106.9)	99.2 (90.7, 104.8)
**cBP sys (mmHg)**	113.7 (104.2, 120.7)	112.8 (104.8, 120.3)
**cBP dia (mmHg)**	82.6 (69.3, 93.1)	85.0 (76.9, 90.9)
**PWV (m/s)**	5.56 (5.19, 5.88)	5.43 (5.11, 5.96)
**Aix@75 (%)**	17.5 (13.1, 20.8)	16.1 (13.5, 21.5)
**AugP (mmHg)**	4.22 (3.66, 7.13)	4.50 (3.00, 6.75)
**Energy Drink (240 mg caffeine, 750 mL)**		
	**Energy Drink**	
	**Before**	**After**
**HR (bpm)**	78.0 (67.5, 83.2)	73.3 (67.8, 78.7)
**pBP sys (mmHg)**	120.8 (114.8, 126.5)	127.4 (120.0, 133.5)
**pBP dia (mmHg)**	76.8 (69.8, 82.6)	82.0 (74.5, 90.0)
**pBP mean (mmHg)**	93.5 (86.5, 102.5)	101.3 (95.8, 105.8)
**cBP sys (mmHg)**	110.4 (102.2, 115.3)	114.7 (109.8, 125.8)
**cBP dia (mmHg)**	77.6 (72.0, 84.6)	84.4 (76.5, 93.4)
**PWV (m/s)**	5.39 (5.04, 5.92)	5.62 (5.24, 6.21)
**Aix@75 (%)**	15.2 (9.0, 23.3)	18.5 (12.0, 26.0)
**AugP (mmHg)**	4.20 (2.50, 8.00)	5.50 (3.00, 10.75)
**Water (0 mg caffeine, 750 mL)**		
	**Water**	
	**Before**	**After**
**HR (bpm)**	74.5 (67.7, 76.4)	66.8 (62.7, 69.1)
**pBP sys (mmHg)**	112.6 (105.1, 126.7)	119.6 (113.0, 131.5)
**pBP dia (mmHg)**	74.9 (65.5, 101.9)	79.2 (75.1, 87.8)
**pBP mean (mmHg)**	89.9 (81.4, 111.8)	94.3 (91.4, 105.1)
**cBP sys (mmHg)**	105.7 (98.2, 122.6)	111.0 (107.5, 118.7)
**cBP dia (mmHg)**	76.2 (66.8, 103.0)	79.9 (76.3, 91.3)
**PWV (m/s)**	5.92 (5.41, 6.73)	5.96 (5.58, 6.96)
**Aix@75 (%)**	14.4 (4.2, 28.9)	12.8 (7.5, 25.3)
**AugP (mmHg)**	4.00 (1.28, 9.50)	4.13 (2.74, 10.13)

All values are presented as median (interquartile range, IQR). HR, heart rate; bpm, beats per minute; p, peripheral; c, central; sys, systolic; dia, diastolic; BP, blood pressure; PWV, pulse wave velocity; Aix@75, augmentation index corrected for a HR of 75 bpm; AugP, augmentation pressure.

**Table 3:** Absolute changes of hemodynamic parameters after caffeine consumption.

	Coffee	Energy Drink	*p*-Value	*p*-Value (adj.)
**HR (bpm)**	−2.28	−4.60	0.166	0.516
**pBP sys (mmHg)**	−0.91	+6.35	**0.013**	0.131
**pBP dia (mmHg)**	+1.00	+4.00	0.276	0.575
**pBP mean (mmHg)**	−0.43	+5.25	**0.016**	0.145
**cBP sys (mmHg)**	−1.63	+7.00	**0.017**	0.145
**cBP dia (mmHg)**	+0.85	+3.07	0.248	0.575
**PWV (m/s)**	−0.04	+0.17	**0.042**	0.257
**Aix@75 (%)**	−0.78	+1.40	0.098	0.421
**AugP (mmHg)**	−0.23	+0.95	0.087	0.421

All values are presented as median. *p*-values were calculated by paired-sample Wilcoxon tests and corrected for multiple comparisons by using the Holm–Sidak method. HR, heart rate; bpm, beats per minute; p, peripheral; c, central; sys, systolic; dia, diastolic; BP, blood pressure; PWV, pulse wave velocity; Aix@75, augmentation index corrected for a HR of 75 bpm; AugP, augmentation pressure; adj., adjusted.

## Data Availability

The data presented in this study is largely available within the article. Additional detailed material is available on request from the corresponding author.
